# Identifying Cancer Drivers Using DRIVE: A Feature-Based Machine Learning Model for a Pan-Cancer Assessment of Somatic Missense Mutations

**DOI:** 10.3390/cancers13112779

**Published:** 2021-06-03

**Authors:** Ionut Dragomir, Adnan Akbar, John W. Cassidy, Nirmesh Patel, Harry W. Clifford, Gianmarco Contino

**Affiliations:** 1 Institute of Cancer and Genomic Sciences, College of Medical and Dental Sciences, University of Birmingham, Edgbaston, Birmingham B15 2TT, UK; ixd935@alumni.bham.ac.uk; 2 Centre for Computational Biology, College of Medical and Dental Sciences, University of Birmingham, Edgbaston, Birmingham B15 2TT, UK; 3 Cambridge Cancer Genomics, Cambridge CB2 1QN, UK; adnan.akbar@open.ac.uk (A.A.); john@ccg.ai (J.W.C.); nirmesh@ccg.ai (N.P.); harry@cancergenomics.co.uk (H.W.C.); 4 Von Hügel Institute, St Edmund College, University of Cambridge, Cambridge CB3 0BN, UK; 5 Queen Elizabeth Hospital, University of Birmingham Hospital Trust, Edgbaston, Birmingham B15 2GW, UK

**Keywords:** pan-cancer, classification, decision tree, driver mutation, extreme gradient boosting, k-nearest neighbours, logistic regression, multilayer perceptron, random forest, support vector machines

## Abstract

**Simple Summary:**

Genes dictate the grounds of life by comprising molecular bases which encode proteins. A mutation represents a gene modification that may influence the protein function. Cancer occurs when the mutation triggers uncontrolled cellular growth. Judging by the cancer expansion, mutations labelled as drivers confer a growth advantage, while passengers do not contribute to this augmentation. The aim of this study is methodological, which assesses the usefulness of a classification method for distinguishing between driver and passenger mutations. Based on 51 molecular characteristics of mutations and genes, including 3 novel features, multiple machine learning algorithms were used to determine whether these characteristics biologically represent the driver mutations and how they impact the classification procedure. To test the ability of the present methodology, the same steps were applied to an independent dataset. The results showed that both gene and mutation level characteristics are representative of the driver mutations, and the proposed approach achieved more than 80% accuracy in finding the true type of mutation. The evidence suggests that machine learning methods can be used to gain knowledge from mutational data seeking to deliver more targeted cancer treatment.

**Abstract:**

Sporadic cancer develops from the accrual of somatic mutations. Out of all small-scale somatic aberrations in coding regions, 95% are base substitutions, with 90% being missense mutations. While multiple studies focused on the importance of this mutation type, a machine learning method based on the number of protein–protein interactions (PPIs) has not been fully explored. This study aims to develop an improved computational method for driver identification, validation and evaluation (DRIVE), which is compared to other methods for assessing its performance. DRIVE aims at distinguishing between driver and passenger mutations using a feature-based learning approach comprising two levels of biological classification for a pan-cancer assessment of somatic mutations. Gene-level features include the maximum number of protein–protein interactions, the biological process and the type of post-translational modifications (PTMs) while mutation-level features are based on pathogenicity scores. Multiple supervised classification algorithms were trained on Genomics Evidence Neoplasia Information Exchange (GENIE) project data and then tested on an independent dataset from The Cancer Genome Atlas (TCGA) study. Finally, the most powerful classifier using DRIVE was evaluated on a benchmark dataset, which showed a better overall performance compared to other state-of-the-art methodologies, however, considerable care must be taken due to the reduced size of the dataset. DRIVE outlines the outstanding potential that multiple levels of a feature-based learning model will play in the future of oncology-based precision medicine.

## 1. Introduction

Sporadic cancer develops from the accrual of somatic mutations that affect the physiological function of genes [1]. Somatic mutations can be divided by size into small-scale and large-scale somatic mutations. The largest proportion of small-scale somatic mutations include point mutations, substitutions, inversions, insertions and deletions. If the point mutations occur inside the coding region of the genome, they can be further categorised as synonymous—which are silent, normally resulting in the same amino acid being produced and nonsynonymous—which can result in a different amino acid or other abnormalities affecting the protein structure [2,3]. Nonsynonymous mutations can be additionally partitioned into missense mutations, in which a different amino acid being produced may impact the protein function, thus resulting in a pathogenic effect, and account for the majority of the nonsynonymous mutations, nonsense mutations, where a stop or nonsense codon is produced and essential splice site mutations, in which a change occurs in the site where the splicing takes place [3,4]. Exploring missense mutations is of utmost importance, since they account for more than 90% of the base substitutions present in the genome coding regions [2].

The completion of large scale genome sequencing projects including The Cancer Genome Atlas (TCGA) and the International Cancer Genome Consortium (ICGC) has fostered the development of multiple bioinformatics algorithms for identifying the role of somatic mutations in cancer [5,6]. Positive selection is the main evolutionary force in cancer, leading to the accumulation of driver mutations in critical genes responsible for tumour proliferation [3]. In contrast, passenger mutations are somatic mutations which occur during cell division but have no functional consequence. The main challenge remains the classification of mutations into driver mutations, which give a fitness advantage to the cell and are under selective pressure and passenger mutations [7,8]. Cancer genomes are also under the selective pressure of negative selection (or purifying selection) that suppresses mutations that are counterproductive for tumour growth (e.g., by eliciting immune response or synthetic lethality) [3]. It was recently suggested that negative selection may play a much more substantial role than was previously estimated [9]. A robust approach has applied the ratio between nonsynonymous and synonymous substitutions in different types of tumours to predict patterns of selection and catalogue cancer genes [3].

Missense mutations have also been shown to selectively target protein–protein interactions (PPIs) in cancer, possibly contributing to the tumour heterogeneity; therefore, protein interaction interfaces may reveal new processes by which positive selection occurs in cancer [10]. However, this particular area has been overlooked in recent analyses despite its potential clinical relevance. Previous work had focused on generating statistical models which are based on the frequency of the mutations, which are expected to occur at a much higher rate compared to the background [11]. While this methodology generated valuable insights by finding common drivers, because it is based on a gene-level investigation, it is often impractical in finding rare driver mutations, since the long-tail hypothesis suggest that cancer driver mutations include few frequent drivers and more unusual ones [12]. Therefore, a supervised feature-based learning methodology is generally regarded as the most practical, since it can be used to extract insights from a biological feature perspective using modern computational resources, while not being influenced by the drawbacks of statistical methods. Nevertheless, distinguishing between a driver and a passenger mutation still presents a classification challenge, due to a lack of a standard definition for a driver mutation and no standard class labels that could be used for machine learning tasks.

The primary purpose of this study is methodological, aiming towards developing a new machine learning approach for driver identification, validation and evaluation (DRIVE) that can predict if a somatic mutation is a driver or a passenger, based on features at both gene and mutation levels integrating molecular characteristics derived from PPIs and pathway analysis. Since passenger mutations are also found in well-established driver genes [2], it has been suggested that more specificity could be achieved by shifting the analysis from a gene perspective to a mutation level [13], without completely eliminating the gene-level characteristics. Within the framework of these criteria, both level features—including novel gene features such as the maximum number of PPIs, the biological process it belongs to and the type of post-translational modifications (PTMs) that the protein product is undergoing will be collected in an attempt to understand if they are a true representation of the driver mutations. Ultimately, the integrative analysis of those features within a machine learning framework may help to identify novel rare driver mutations and discover patterns for patient-specific drivers that can be exploited for targeted therapeutics.

## 2. Methods

**Figure 1:**
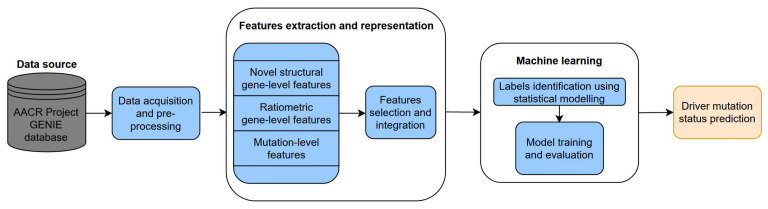
Overview of the main steps of the current approach.

A simplified diagram of the main computational steps can be seen in Figure 1, while a more detailed overview including all the pre-processing stages can be seen in Supplementary Figure S1: Detailed overview of the computational workflow.

### 2.1. Data Acquisition and Pre-Processing

The pan-cancer mutation dataset was obtained from the Genomics Evidence Neoplasia Information Exchange (GENIE) project (v6.0, variants having been aligned corresponding to GRCh37 build of the reference genome), which is supported by the American Association for Cancer Research (AACR) and employs 19 cancer centres on an international level [14]. It is currently the largest fully public registry of cancer, aiming to provide high-quality mutational data based on gene panels, which are considered highly relevant within 80 major types of cancer. The original Mutation Annotation Format (MAF) file—initially created for the TCGA project—comprises multiple types of somatic alterations along with other descriptors which can be found in the GENIE data guide [15,16]. The sequencing strategy involves targeting gene panels including hotspot regions and all exons coverage. Since the type of analysis is pan-cancer based, all samples were considered and not filtered by the cancer type. Additionally, due to the high-quality content of the GENIE dataset, all the mutations are already passing all quality filter parameters.

### 2.2. Feature Extraction

The current approach utilises 51 features at both gene and mutation levels. A detailed description of each feature, as well as the original source, can be found in the Supplementary Table S1: Features, detailed description and the original data source.

#### 2.2.1. Gene-Level Features

The structural features include 3 broad categories. Firstly, the maximum number of PPIs was extracted from the Integrated Structural Interactome and Genomic Data Browser (Interactome INSIDER). The highest confidence interfaces include either interactions based on their Protein Data Bank (PDB) structure, homology models or potential interfaces from the Ensemble Classifier Learning Algorithm to predict the Interface Residues (ECLAIR) algorithm. ECLAIR is a machine learning framework that predicts interactions according to biophysical, structural and docking-based metrics, having an estimated precision of 0.69 for high and very high confidence interfaces [17]. Secondly, the biological process category to which the gene sets used for the PPIs interfaces belong to were extracted from MSigDB (v7.1) using a msigdbr R package based on gene set enrichment analysis (GSEA) [18,19]. The enrichment was performed with a 0.05 *p*-value cutoff using the Benjamini–Hochberg adjustment method for multiple comparisons and a q-value cutoff of 0.05 [20]. The minimum gene set size for the enrichment was set to 20, while the maximum was agreed at 500 based on literature evidence that a very small set size would include redundant pathways, while a very large gene set size would lead to an overly imprecise result [21]. This particular feature which comprises 9 biological processes (which can be expanded to 50 hallmarks) was selected, since there is evidence of driver mutations triggering certain molecular pathways. Nonetheless, these processes present different properties due to tumour heterogeneity, suggesting that the mutation patterns can help identify which pathway events are linked to causing cancer [22]. Thirdly, the types of PTMs a certain protein undergoes were extracted from PhosphoSitePlus (v6.5.9.2). This database comprises PTMs mostly determined from new mass spectrometry data, or having been previously determined using at least 1,000,000 spectra [23]. Throughout the use of PTM data, it was revealed that regions prone to lysine modifications showed a critical functional impact in driving cancer and a potential tool for treatment or diagnosis [24], therefore taking these modifications into account was geared towards their potential new insights into cancer characterisation based on specific PTMs. Ratiometric features include characteristics based on the structure of the mutations which were normalised by the total mutations in a specific gene. These features were already extracted from the 20/20+ study founded on a simplification of the 20/20 rule, which is based on a decision tree that classifies genes into oncogenes, tumour suppressor genes and passengers with regards to the threshold scores [25]. They comprised multiple categories, including the length of the reference transcript, the fraction of silent mutations, nonsense mutations proportion, splice site mutations or recurrent missense mutations, ratios of missense to silent mutations and non-silent to silent mutations. Furthermore, these scores include the gene prediction of being an oncogene, tumour suppressor gene or a passenger which was generated using a random forest approach.

#### 2.2.2. Mutation-Level Features

Features on a mutation-level were computed using the web interface of Variant Effect Predictor (VEP) based on Ensembl genome database (release 100, April 2020), which annotates genomic variants based on their consequence [26]. Sorting Intolerant From Tolerant (SIFT) and Polymorphism Phenotyping (PolyPhen) scores were chosen, as they show the potential impact of amino acid substitutions on the protein function [27,28], while Consensus Deleteriousness (Condel) scores indicate the probability of a single base mutation of being deleterious [29]. Thus, a SIFT score of 0 represents the most deleterious variant while a PolyPhen or a Condel score of 1 shows the variant with the highest deleterious potential. Additional rank scores were extracted from the database for Nonsynonymous SNPs’ Functional Predictions (dbNSFP), which provides pathogenicity scores from multiple popular algorithms [30]. The highest rank score for each mutation was chosen and then the average value between all scores was calculated based on the rationale that the highest rank score will show the potentially most damaging effect, while the arithmetic mean will reflect the overall pathogenic consequence. A full list of all rank scores used for the computation can be found in Supplementary Table S2: List of features used to compute the average rank score.

### 2.3. Labels Compilation Using Statistical Modelling

Considerable care must be taken when generating the true class labels, since there is no standard protocol for generating driver mutations labels, due to a lack of a universal definition for what is regarded as a driver mutation. Therefore, the labels were extracted from a frequency-based model study, which categorised the mutations as drivers or passengers based on mutational hotspots [31]. During this study, it was determined that more than half of the analysed tumours have a mutational hotspot, affecting at least 275 protein-coding genes. These regions are defined as specific positions of the amino acids included in a gene that encodes a protein that suffers point mutations more frequently than in the absence of the positive selection. The statistical significance is based on a binomial probability including the mutation rate in cancer and patterns of mutational selection which present gene specificity. This labelling system was regarded as the best procedure for the proposed methodology, since it comprises hotspots not only at a whole-genome level but encompassing a pan-cancer magnitude considered critical for the purpose of this analysis.

### 2.4. Feature-Based Learning and Performance Evaluation

#### 2.4.1. Addressing the Class Imbalance Problem

The initial dataset was imbalanced, with labels for driver mutations occurring in a much less number compared to the passenger mutations. To address this problem, undersampling was used, which included removing random rows from the majority class until both classes have the same number of occurrences [32]. Although this method suffers from losing important data, it was preferred, since the alternative solution is oversampling, which can be prone to overfitting.

#### 2.4.2. Pre-Processing

For all models, to account for variation due to different datasets being merged, scaling was applied as a pre-processing step which subtracts the mean value and then divides by the standard deviation, therefore transforming the mean to 0 and the standard deviation to 1.

#### 2.4.3. Resampling Method

K-fold cross-validation (k = 10) was used as the resampling procedure, based on the fact that it is known to reduce selection bias and overfitting by retaining one subsample as the validation data, while the other k − 1 subsamples are used for training [33]. Then, the procedure is repeated k times until each subsample is used for validation.

#### 2.4.4. Training

All models were trained using the caret R package and its dependencies for supervised learning methods including random forest, decision tree, extreme gradient boosting (EGB), support vector machines (SVM), k-nearest neighbours (KNN), logistic regression and multilayer perceptron (MLP) [34]. A full list of all required packages can be found on the GitHub repository. At this stage of the investigation, pruning techniques have not been applied, so that all features were considered for assessing the ranked feature importance for the most powerful classifier at a later point of the analysis.

#### 2.4.5. Hyperparameters Optimisation

Hyperparameters optimisation steps were included automatically in the train function of the caret package, where 10 random values were used for each parameter of each model. At last, the values which showed the best performance adjudicating by the receiver operating characteristics (ROC) metric were retained as the final model. A list of the final hyperparameters chosen, as well as a brief description of each parameter, can be found in Table S3: Final hyperparameters values and description.

#### 2.4.6. Performance Evaluation Measures

The performance was evaluated using multiple metrics including accuracy, precision, recall, F1-score, and area under the curve of a ROC curve (AUC-ROC), which were computed using a confusion matrix between the predicted and the true class labels [35].

#### 2.4.7. Feature Importance

To assess the feature weight of the classifier that showed the finest performance, feature importance was computed using the mean decrease in accuracy (MDA) and the mean decrease in impurity (MDI), using the varImp function of the caret package [34]. The former evaluates the importance by removing the link between a specific feature and the target classes while applying permutations and returning the increase in the error rate. The latter calculates feature importance as the total number of occurrences of a feature throughout all splits, however, it is also known that this importance weighting method suffers from biases arising from multiple testing inside the splits since variables where more splits are applied tend to appear more often inside the tree than is expected by chance [36].

### 2.5. Testing on an Independent Dataset

On the grounds of the model being trained only on the dataset presented above, it was decided that the best approach would be to predict the results on an independent dataset and further assess the performance. For this purpose, the same data format was extracted from the CHASMplus study, a dataset that belongs to the TCGA project (v0.2.8) and comprises somatic mutation calls commonly referred to as multi-center mutation calling in multiple cancers (MC3) [13]. The same procedure for data pre-processing and feature extraction was followed as stated in steps 1 and 2 of the methodology. After addressing the class imbalance problem, the final dataset comprised 3914 mutations with equal numbers of positive and negative classes. This dataset already included class labels that were based on a semi-supervised approach following three conditions [13]. Firstly, the mutation needed to occur in a curated set of 125 pan-cancer established genes [2]. Secondly, for a specific type of cancer, the mutation ought to be in a significantly mutated gene for that cancer type [37]. Thirdly, the mutation had to take place in an area with a mutation rate under 500 mutations, which has been defined as a hypermutator threshold. A critical point to consider at this stage is that this semi-supervised procedure used for labels follows a cluster adherence assumption based on the rationale that driver mutations occur in well-known significantly mutated driver genes for specific cancer types [13]. Therefore, all mutations fulfilling the aforementioned conditions were classified as drivers, while the remaining mutations received a passenger label.

### 2.6. Performance Evaluation on the Benchmark Dataset

For assessing the performance of the highest-scoring classifier, a benchmark dataset provided by the MutaGene computational framework was used [38]. This dataset comprises predictions for each mutation of being a potential driver or potential passenger, which were assembled from various experimental studies of multiple mutational patterns derived from large-scale projects such as TCGA and ICGC, which benefit from being corrected for biases arising from mutational hotspots or background DNA mutation rates. The combined annotation was mainly based on the Catalogue Of Somatic Mutations In Cancer (COSMIC, v75), classifying mutations into neutral and non-neutral [39], thus for the present evaluation, these represented the true class labels. Performance assessment was done using the random forest model to predict on the benchmark dataset, while also using CHASMplus (v1.2.0) through Open Custom Ranked Analysis of Variants Toolkit (OpenCRAVAT) web server for extracting the mutation probability of being a driver [40].

## 3. Results

Training data were collected from the GENIE project [14], and after filtering comprised 357,778 missense mutations across 59,692 samples. Based on the evidence that missense mutations can target PPIs [10], the maximum number of high-quality interactions for each protein were extracted from the computationally curated Interactome INSIDER structural database ranging from 1 to 290 interactions [17]. This feature was chosen because it has been shown that missense mutations linked to cancer can target PPIs, conceivably contributing to tumour heterogeneity [10]. For investigating the impact of biological processes disturbed in cancer [22], 1390 groups of genes linked by known interactions were extracted from MSigDB, comprising 9 non-redundant biological processes which were extracted based on the GSEA [18,41] and an additional number of 50 molecular signatures which could extend the analysis to gene-specific pathways. A number of 13 different PTMs occurring on 9 types of residues were manually extracted from PhosphoSitePlus, based on the rationale that certain amino acid residues such as lysine are specifically affected in driver genes [24]. Based on the 20/20+ study, an additional number of 24 ratiometric features were added to the dataset, since previous research has shown that ratios such as missense to silent mutations may play a distinctive role in driver mutation classification [25]. Finally, VEP was used to extract mutation-level features including SIFT, PolyPhen and Condel rank scores. Moreover, the computed average based on other pathogenicity scores was added to the mutation-level features based on the rationale that this feature may improve driver detection by eliminating the false positives arising due to driver gene labelling [13,42]. After adding the labels based on mutational hotspots, undersampling was used for addressing the imbalanced class problem. Therefore, an equal number of 12,297 of both driver and passenger mutations was used, since an imbalanced dataset can show discrepancies between different iterations and it is also prone to overfitting [32]. Features at both levels were collected, each level being expected to reveal distinct molecular characteristics which could potentially be exploited in the context of cancer driverness. The current approach utilises a total number of 51 features across gene and mutation levels. On the final dataset, multiple state-of-the-art supervised machine learning algorithms were applied, of which the results from the ones with the highest performance were presented including random forest, logistic regression, EGB, KNN, SVM, logistic regression and MLP.

### 3.1. Performance Evaluation of the Machine Learning Models

For each machine learning approach, a set of 10 hyperparameter combinations, which are model parameters set before starting the machine learning procedure, were used to determine the best variables. The full set of hyperparameters values and their description can be found in Supplementary Table S3. Thus, by resampling each combination, estimates for the performance measure were extracted and those corresponding to the best outcome were used for the 10-fold cross-validation learning process. For each final model, the confusion matrix was used to extract the performance metrics which show the average performance across all folds. As it can be seen in Table 1, from all models, the random forest has the best AUC-ROC, while the decision tree model shows the best performance based on the other performance metrics. Broadly speaking, random forest, logistic regression and decision trees show better performance compared to the other models. Nevertheless, the MLP classifier did not present an adequate performance for this type of classification problem compared to the other ensemble methods. Multiple variants of MLP were trained on the same dataset, but their performance was significantly lower, therefore due to the nature of the data, it was considered that this category of artificial neural networks was not suitable for this classification purpose.

**Figure 2:**
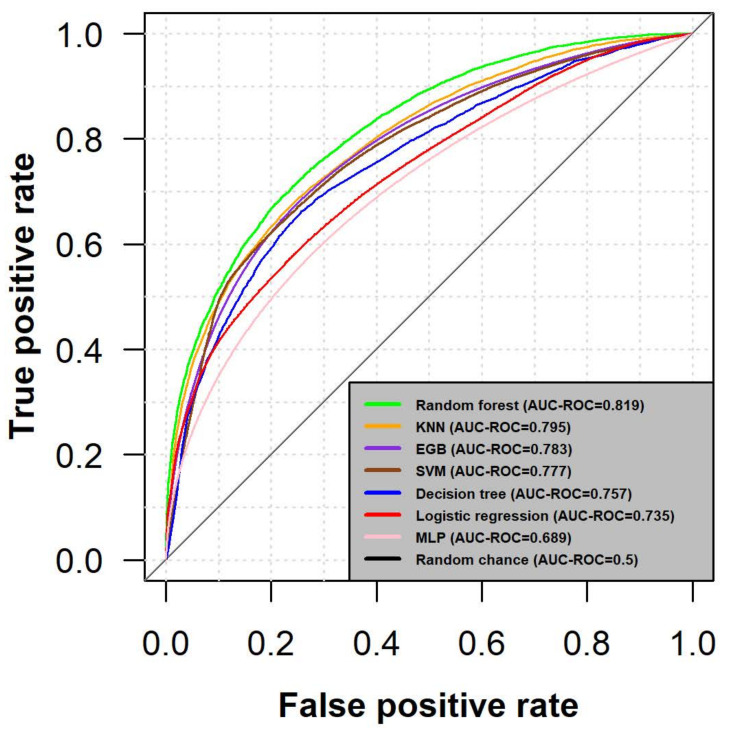
Performance evaluation using the average AUC-ROC of 10-fold cross-validation. The abscissa reflects the False Positive Rate (FPR) and displays a function of expressing a Type I error by which a mutation is classified as a driver when in fact it is a passenger. The ordinate shows the true positive rate (TPR) and reflects the prospective rate of detecting a positive signal when it is a real positive, thus classifying a real driver mutation as a driver. A perfect classification model is deemed as approaching the top right corner with an AUC-ROC = 1, while a random classifier is generally regarded as finding the class due to random chance (AUC-ROC = 0.5) and is represented by the diagonal line.

For a better visual representation of the true positive rate (TPR) over the false positive rate (FPR), the ROC curves for each model were plotted using pROC R package [43], as can be seen in Figure 2. Random forest classifier indicates the best performance (AUC-ROC = 0.819) which is regarded as in almost 82% of the cases, the model will correctly assign a driver to its true label category.

### 3.2. Feature Importance of Random Forest

**Figure 3:**
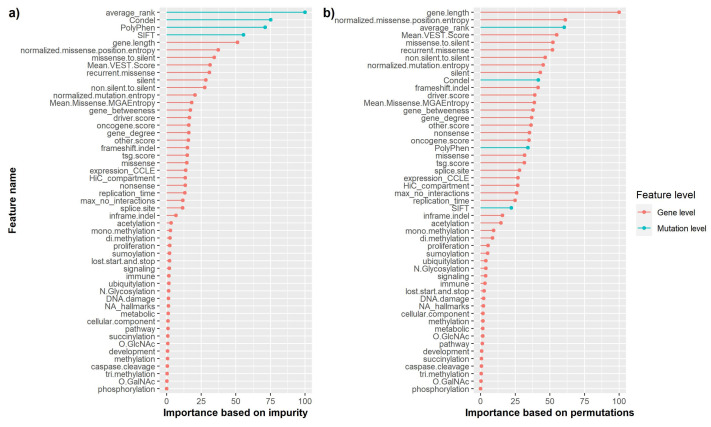
Feature importance of the random forest classifier across all 51 features. The feature significance was addressed using two methods. Firstly, the importance was calculated based on the MDI (**a**). Secondly, the MDA was used (**b**) which is regarded as a method that is less prone to biases arising due to multiple testing [36]. All importance levels were scaled on a 0-100 range, with 0 representing the least important feature, while 100 signifies a vital characteristic.

Since the performance of random forest was considered the most significant in the proposed approach based on the AUC-ROC metric, feature importance was computed for assessing the impact of each independent feature within the full set of 51 features, as well as the overall impact of both feature levels. The results based on the MDI and MDA can be observed in Figure 3. Since there is evidence that MDI biases may be influenced by multiple testing, feature significance was also computed based on MDA, from which it can be seen that not only gene-level features are of utmost importance, but also mutation-level features.

The most significant gene-level features highlighted by the current methodology are the gene length, the measure of the positional clustering, the mean Variant Effect Scoring Tool (VEST) score and the ratio of missense to silent mutations. From a mutation-level perspective, the average across all rank scores shows great importance, while other features are not regarded as significant based on the permutations metric method.

### 3.3. Prediction on an Independent Dataset

**Figure 4:**
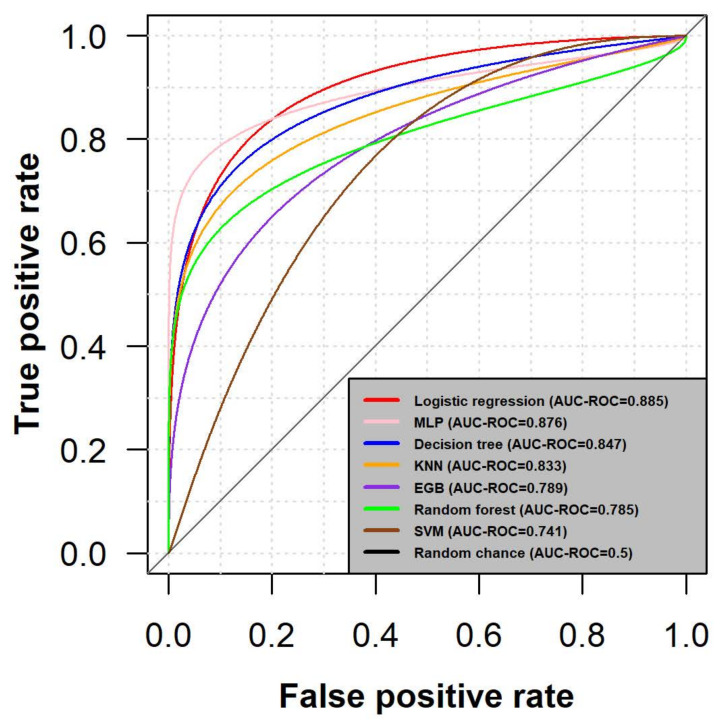
Prediction assessment on an independent dataset. Based on the grounds that the GENIE project used a targeted sequencing approach [14], the model was additionally validated on a subset of TCGA data. The true class labels within this dataset were generated using a semi-supervised approach based on a cluster adherence assumption [13]. Due to the initial shape of the ROC curves, a binormal smoothing procedure was applied based on the presumption that both positive and negative classes are normally distributed, therefore following a linear relationship defined by a regression curve [43].

The training dataset includes sequencing data from the GENIE project, which used all exons or mutational hotspots as sequencing strategies, depending on the research institution and the cancer-causing genes [14]. The gene panels are focused on the most mutated genes with known oncogenic potential [44], therefore this may affect the performance of the model due to the selection of different variants and the influence of the sequencing technique. Thus, the model was further validated on an independent dataset that comprised whole-exome data. As can be seen in Table 2, logistic regression had the best overall performance across all metrics, including a remarkably high value for AUC-ROC. Before interpreting the results, it is important to mention that the true class labels were obtained using a different approach than in the training dataset. For visually inspecting the performance, a plot of the AUC-ROC metric of each model prediction can be observed in Figure 4.

The results indicate that the best model of the prediction on the new dataset (having an AUC-ROC of 0.885) will accurately allocate a driver mutation to its genuine category in almost 90% of the cases.

Apart from a slight discordance with regards to the best performing algorithm and a different approach used in generating the true class labels, the result is a confirmation of the good representation of driver mutations by both gene and mutation-level features. Of the newly extracted features, the maximum number of PPIs appears to play the most important role, accounting for 25% of the overall importance. However, it shall be considered that only high-quality based interactions were extracted, therefore their contribution might be higher than it can be observed if medium confidence intervals were added. The biological process presents modest importance, however, a new analysis including all 50 molecular signatures may offer a better characterisation of the driver mutations. PTMs also show moderate importance and this could be attributed to the fact that the general PTMs did not include the specific residues and only the general modification type. Nevertheless, if all the aforementioned steps would have been added to the training procedure, a higher computational power, as well as a much higher analysis time, need to be considered.

### 3.4. Performance Evaluation on the Benchmark Dataset

**Figure 5:**
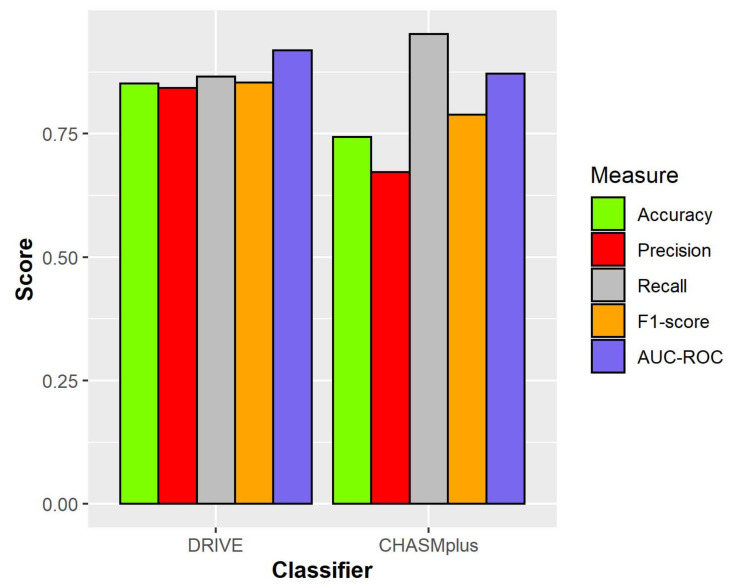
Visual representation of the performance metrics after using DRIVE and the current state-of-the-art model (CHASMplus). However, extreme caution must be exercised when interpreting the results, since the size of the benchmark dataset was limited.

The initial benchmark dataset was provided by the MutaGene mutation analysis tool and comprised an initial set of 5276 mutations. Nevertheless, since the mutations did not include genomic coordinates, these were extracted using TransVar annotator (v2.4.0) [45]. Consequently, all features were extracted again using the above-mentioned methodology, comprising 4244 mutations with a complete feature set. These mutations were inputted into CHASMplus (v1.2.0) classifier via OpenCRAVAT web server [40], this approach being considered one of the best scoring state-of-the-art models which has outclassed other methodologies [13]. The output comprised 1578 mutations and a score that represents the probability of a mutation of being a driver. Moreover, to extract the performance measures for the comparison between these approaches, the random forest model was used to predict driver mutations in the final benchmark dataset. Then, the probabilities based on the CHASMplus model were generated using the hmeasure R package for extracting the performance scores using a 0.5 threshold [46]. A raw comparison can be seen in Table 3, showing that overall, DRIVE scored higher than CHASMplus, while a better visual comparison is represented in Figure 5. Given that our results are based on a limited number of mutations with the full feature set, the results from this analysis should therefore be treated with considerable caution.

## 4. Discussion

Cancer genomics studies still face an ongoing challenge due to the presence of several types of mutations occurring with different phenotypic effects. Substitution mutations account for 95% of all somatic mutations, out of which 90% are missense mutations [2]. Mutations can be further divided into driver and passenger mutations based upon the fitness advantage conferred to the cancer growth. The emergence of driver mutations as well as cancer heterogeneity are key elements in overcoming treatment resistance and failure [47,48].

Although previous approaches focused on the frequency of mutations, this study is based on incorporating mutation-level and gene-level features by using a supervised machine learning method to determine if the features are representative of the driver mutations and if they can be further used to detect new drivers. The results indicated that ensemble learning methods perform better than other models, random forest achieving the most striking performance (with an accuracy of 0.734 and AUC-ROC of 0.819). This demonstrates that the proposed features are a good representation of the driver mutations, thus providing a powerful tool for drivers detection which is not limited to only identifying the most common ones. The feature importance based on the mean decrease in accuracy shows that the most important feature on a gene-level is the gene length. The current findings appear to be well supported by the DriverFinder study, which states that variants tend to occur more in longer genes [49], yet this study is concentrated on driver genes and not driver mutations. Therefore, there is a high chance of including false positive driver mutations during the extraction phase based on driver genes, which are regarded as being a driver for the reason that they are inside a driver gene [8]. As anticipated, the mutation-level features also present an important role, the average rank showing substantial importance. There is a satisfactory agreement of this result and description of the dbNSFP, since they are expected to show critical pathogenic consequences, as the dataset is based on a pan-cancer analysis [30].

Further prediction using DRIVE on an independent dataset reinforces the usefulness of a feature-based learning method achieving a better performance compared to the training dataset (where the logistic regression classifier had an accuracy of 0.781 and an AUC-ROC of 0.885). However, given that these findings are based on a different method of generating class labels, the results should be therefore treated with considerable caution, since categorising mutations based on gene scores could be prone to false positives. Despite these shortcomings, the findings are still substantial, since the independent dataset was generated using whole-exome sequencing and not gene panels based on the most relevant genes within 80 major cancers, thus having the potential of new driver mutations discovery. The evaluation of DRIVE on the benchmark dataset and the comparison with the CHASMplus state-of-the-art model show that the current approach has a comparable overall performance. Nevertheless, the small size of the final benchmark dataset compared to the training data size needs to be taken into account, since overfitting may be plausible. Despite this small shortcoming, the current methodology is still regarded as a potential clinical tool that is expected to be used in a clinical oncology setting in the near future.

The present study has only investigated the status and the effect on the cancer growth of somatic missense mutations, therefore the interpretation of the findings was limited in several ways. The first limitation is that DRIVE does not predict clinical outcomes. In fact, drug response and progression may be affected by different subclones conferring treatment resistance. The second limitation is based on the interplay between driver and passenger mutations. It has been suggested that due to environmental conditions and selection type, driver mutations can switch to becoming passenger mutations [50]. Thus, there is some likelihood that this classification may only show the real status of a mutation at a given time and not a coherent whole of mutation characterisation, which can be applied from personalised medicine from beginning to the end of the cancer treatment. The third limitation is that given the limited size of the benchmark dataset, caution must be exercised when interpreting the comparison results between DRIVE and CHASMplus. Therefore, further validation on a larger dataset has to be demonstrated for the final validation of DRIVE.

Further work will concentrate on improving the approach, particularly by adding more features, since there is evidence that certain protein domains present in tumour suppressor proteins tend to suffer mutations in specific domains [51] and it is expected that these additional features are likely to improve the performance of DRIVE. Updating the benchmark dataset so that its size will increase, as well as matching it according to the most recent version of COSMIC represent further tasks that will need to be undertaken. Other features which may potentially improve the classification performance include how driver mutations affect the enzyme function, as well as the protein–DNA interactions and intracellular location of the protein. Additionally, the prospect of being able to apply DRIVE on new cancer datasets and potentially discover new driver mutations serves as a continuous incentive for providing an application programming interface (API) for general public use. This API will benefit from being able to take mutations as the input, while the DRIVE pipeline will extract features at both mutation and gene levels and predict the status of the mutations.

## 5. Conclusions

This study emphasised the valuable insights which can be gained using DRIVE, a feature-based learning approach for distinguishing between driver and passenger mutations. The results suggest that not only do gene-level features play a critical role in being representative for the driver mutations, but mutation-based features which seem to improve the characterisation of the mutational pathogenic impact also have a large part to play. Furthermore, the current model showed a better performance on the limited size of the benchmark dataset than other state-of-the-art models, suggesting the importance of including features at multiple levels. Taken together, a machine learning approach represents a better alternative for distinguishing between driver and passenger mutations compared to classic frequency-based models, aiming towards the final goal of delivering a more efficient personalised treatment and improving the patient’s outcome.

## Figures and Tables

**Table 1:** Performance evaluation of training the supervised classification models. The performance metrics (accuracy, precision, recall, F1-score and AUC-ROC) show how well each classifier performed using the full set of 51 features. Based on AUC-ROC, the random forest model had the leading performance after 10-fold cross-validation and hyperparameter optimisation. A default threshold of 0.5 was used for computing the two-class summary. A detailed description of each hyperparameter (such as the randomly selected predictors, splitting rule and the minimal node size for the random forest classifier) can be found in Supplementary Table S3. The R code used to produce the performance metrics and the hyperparameter values can be found in the GitHub repository: https://github.com/ccgenomics/DRIVE-ML (accessed on 24 April 2021).

Algorithm	Accuracy	Precision	Recall	F1-Score	AUC-ROC
Random forest	0.734	0.741	0.718	0.729	0.819
KNN	0.414	0.435	0.570	0.493	0.795
EGB	0.659	0.971	0.327	0.489	0.783
SVM	0.478	0.483	0.614	0.541	0.777
Decision tree	0.702	0.724	0.650	0.685	0.757
Logistic regression	0.697	0.739	0.607	0.667	0.735
MLP	0.647	0.713	0.492	0.582	0.689

**Table 2:** Assessment of DRIVE using the prediction on an independent dataset across all 51 features. The performance metrics (accuracy, precision, recall, F1-score and AUC-ROC) indicate that the features spanning multiple biological levels are an adequate representation of the driver mutations. The default threshold of 0.5 was used for computing the two-class summary. The hyperparameters values used, as well as their description, can be found in Supplementary Table S3. The R code which was used to extract the performance metrics and the final hyperparameters values can be found in the GitHub repository.

Algorithm	Accuracy	Precision	Recall	F1-Score	AUC-ROC
Logistic regression	0.781	0.795	0.756	0.775	0.885
MLP	0.786	0.983	0.582	0.731	0.876
Decision tree	0.723	0.723	0.723	0.723	0.847
KNN	0.753	0.750	0.758	0.754	0.833
EGB	0.699	0.687	0.732	0.708	0.789
Random forest	0.706	0.696	0.731	0.713	0.785
SVM	0.680	0.672	0.706	0.688	0.741

**Table 3:** Performance evaluation on the benchmark dataset. The current approach (DRIVE) is compared to the state-of-the-art model (CHASMplus) based on multiple performance metrics, both methodologies having been applied to the MutaGene computational framework. Overall, DRIVE outclassed CHASMplus, however, the limited size of the benchmark dataset has to be taken into consideration.

Model	Accuracy	Precision	Recall	F1-Score	AUC-ROC
DRIVE	0.852	0.843	0.866	0.854	0.919
CHASMplus	0.743	0.672	0.952	0.788	0.872

## Data Availability

Publicly available datasets were analysed in this study. The data, as well as the R code, can be found in the next GitHub repository: https://github.com/ccgenomics/DRIVE-ML (accessed on April 24th 2021).

## Supplementary Materials

The following are available online at https://www.mdpi.com/article/10.3390/cancers13112779/s1, Figure S1: Detailed overview of the computational workflow, Table S1: Features, detailed description and the original data source, Table S2: List of features used to compute the average rank score, Table S3: Final hyperparameters values and description.

## References

[1] Pomerantz M.M., Freedman M.L. (2011). The Genetics of Cancer Risk. Cancer J..

[2] Vogelstein B., Papadopoulos N., Velculescu V.E., Zhou S., Diaz L.A., Kinzler K.W. (2013). Cancer Genome Landscapes. Science.

[3] Martincorena I., Raine K.M., Gerstung M., Dawson K.J., Haase K., Van Loo P., Davies H., Stratton M.R., Campbell P.J. (2017). Universal Patterns of Selection in Cancer and Somatic Tissues. Cell.

[4] Chu D., Wei L. (2019). Nonsynonymous, synonymous and nonsense mutations in human cancer-related genes undergo stronger purifying selections than expectation. BMC Cancer.

[5] Tomczak K., Czerwińska P., Wiznerowicz M. (2015). The Cancer Genome Atlas (TCGA): An immeasurable source of knowledge. Contemp. Oncol. (Pozn).

[6] Zhang J., Baran J., Cros A., Guberman J.M., Haider S., Hsu J., Liang Y., Rivkin E., Wang J., Whitty B. (2011). International Cancer Genome Consortium Data Portal—A one-stop shop for cancer genomics data. Database (Oxford).

[7] Salvadores M., Mas-Ponte D., Supek F. (2019). Passenger mutations accurately classify human tumors. PLoS Comput. Biol..

[8] Stratton M.R., Campbell P.J., Futreal P.A. (2009). The cancer genome. Nature.

[9] Zapata L., Pich O., Serrano L., Kondrashov F.A., Ossowski S., Schaefer M.H. (2018). Negative selection in tumor genome evolution acts on essential cellular functions and the immunopeptidome. Genome Biol..

[10] Engin H.B., Kreisberg J.F., Carter H. (2016). Structure-Based Analysis Reveals Cancer Missense Mutations Target Protein Interaction Interfaces. PLoS ONE.

[11] Dees N.D., Zhang Q., Kandoth C., Wendl M.C., Schierding W., Koboldt D.C., Mooney T.B., Callaway M.B., Dooling D., Mardis E.R. (2012). MuSiC: Identifying mutational significance in cancer genomes. Genome Res..

[12] Ding L., Wendl M.C., Koboldt D.C., Mardis E.R. (2010). Analysis of next-generation genomic data in cancer: Accomplishments and challenges. Hum. Mol. Genet..

[13] Tokheim C., Karchin R. (2019). CHASMplus Reveals the Scope of Somatic Missense Mutations Driving Human Cancers. Cell Syst..

[14] (2017). AACR Project GENIE: Powering Precision Medicine through an International Consortium. Cancer Discov..

[15] Chandran U.R., Medvedeva O.P., Barmada M.M., Blood P.D., Chakka A., Luthra S., Ferreira A., Wong K.F., Lee A.V., Zhang Z. (2016). TCGA Expedition: A Data Acquisition and Management System for TCGA Data. PLoS ONE.

[16] AACR (2020). GENIE Data Guide.

[17] Meyer M.J., Beltrán J.F., Liang S., Fragoza R., Rumack A., Liang J., Wei X., Yu H. (2018). Interactome INSIDER: A structural interactome browser for genomic studies. Nat. Methods.

[18] Liberzon A., Birger C., Thorvaldsdóttir H., Ghandi M., Mesirov J.P., Tamayo P. (2015). The Molecular Signatures Database (MSigDB) hallmark gene set collection. Cell Syst..

[19] Dolgalev I. (2020). Msigdbr: MSigDB Gene Sets for Multiple Organisms in a Tidy Data Format. https://igordot.github.io/msigdbr/.

[20] Benjamini Y., Hochberg Y. (1995). Controlling The False Discovery Rate—A Practical And Powerful Approach To Multiple Testing. J. R. Statist. Soc. Ser. B.

[21] Reimand J., Isser R., Voisin V., Kucera M., Tannus-Lopes C., Rostamianfar A., Wadi L., Meyer M., Wong J., Xu C. (2019). Pathway enrichment analysis and visualization of omics data using g:Profiler, GSEA, Cytoscape and EnrichmentMap. Nat. Protoc..

[22] Leiserson M.D.M., Blokh D., Sharan R., Raphael B.J. (2013). Simultaneous Identification of Multiple Driver Pathways in Cancer. PLoS Comput. Biol..

[23] Hornbeck P.V., Zhang B., Murray B., Kornhauser J.M., Latham V., Skrzypek E. (2015). PhosphoSitePlus, 2014: Mutations, PTMs and recalibrations. Nucleic Acids Res..

[24] Chen L., Miao Y., Liu M., Zeng Y., Gao Z., Peng D., Hu B., Li X., Zheng Y., Xue Y. (2018). Pan-Cancer Analysis Reveals the Functional Importance of Protein Lysine Modification in Cancer Development. Front. Genet..

[25] Tokheim C.J., Papadopoulos N., Kinzler K.W., Vogelstein B., Karchin R. (2016). Evaluating the evaluation of cancer driver genes. Proc. Natl. Acad. Sci. USA.

[26] McLaren W., Gil L., Hunt S.E., Riat H.S., Ritchie G.R.S., Thormann A., Flicek P., Cunningham F. (2016). The Ensembl Variant Effect Predictor. Genome Biol..

[27] Sim N.L., Kumar P., Hu J., Henikoff S., Schneider G., Ng P.C. (2012). SIFT web server: Predicting effects of amino acid substitutions on proteins. Nucleic. Acids Res..

[28] Adzhubei I., Jordan D.M., Sunyaev S.R. (2013). Predicting Functional Effect of Human Missense Mutations Using PolyPhen-2. Curr. Protoc. Hum. Genet..

[29] González-Pérez A., López-Bigas N. (2011). Improving the Assessment of the Outcome of Nonsynonymous SNVs with a Consensus Deleteriousness Score, Condel. Am. J. Hum. Genet..

[30] Liu X., Jian X., Boerwinkle E. (2011). dbNSFP: A Lightweight Database of Human Nonsynonymous SNPs and Their Functional Predictions. Hum. Mutat..

[31] Chang M.T., Asthana S., Gao S.P., Lee B.H., Chapman J.S., Kandoth C., Gao J., Socci N.D., Solit D.B., Olshen A.B. (2016). Identifying recurrent mutations in cancer reveals widespread lineage diversity and mutational specificity. Nat. Biotechnol..

[32] Buda M., Maki A., Mazurowski M. (2018). A systematic study of the class imbalance problem in convolutional neural networks. Neural Netw..

[33] Arlot S., Celisse A. (2010). A survey of cross-validation procedures for model selection. Statist. Surv..

[34] Kuhn M. (2020). Caret: Classification and Regression Training. https://github.com/topepo/caret/.

[35] Hossin M. (2015). A Review on Evaluation Metrics for Data Classification Evaluations. Int. J. Data Min. Knowl. Manag. Process..

[36] Strobl C., Boulesteix A.L., Zeileis A., Hothorn T. (2007). Bias in random forest variable importance measures: Illustrations, sources and a solution. BMC Bioinf..

[37] Lawrence M.S., Stojanov P., Mermel C.H., Garraway L.A., Golub T.R., Meyerson M., Gabriel S.B., Lander E.S., Getz G. (2014). Discovery and saturation analysis of cancer genes across 21 tumor types. Nature.

[38] Goncearenco A., Rager S.L., Li M., Sang Q.X., Rogozin I.B., Panchenko A.R. (2017). Exploring background mutational processes to decipher cancer genetic heterogeneity. Nucleic Acids Res..

[39] Tate J.G., Bamford S., Jubb H.C., Sondka Z., Beare D.M., Bindal N., Boutselakis H., Cole C.G., Creatore C., Dawson E. (2019). COSMIC: The Catalogue Of Somatic Mutations In Cancer. Nucleic Acids Res..

[40] Pagel K.A., Kim R., Moad K., Busby B., Zheng L., Hynes-Grace M., Tokheim C., Ryan M., Karchin R. (2019). OpenCRAVAT, an open source collaborative platform for the annotation of human genetic variation. bioRxiv.

[41] Subramanian A., Tamayo P., Mootha V.K., Mukherjee S., Ebert B.L., Gillette M.A., Paulovich A., Pomeroy S.L., Golub T.R., Lander E.S. (2005). Gene set enrichment analysis: A knowledge-based approach for interpreting genome-wide expression profiles. Proc. Natl. Acad. Sci. USA.

[42] Richards S., Aziz N., Bale S., Bick D., Das S., Gastier-Foster J., Grody W.W., Hegde M., Lyon E., Spector E. (2015). Standards and Guidelines for the Interpretation of Sequence Variants: A Joint Consensus Recommendation of the American College of Medical Genetics and Genomics and the Association for Molecular Pathology. Genet. Med..

[43] Robin X., Turck N., Hainard A., Tiberti N., Lisacek F., Sanchez J.C., Müller M. (2011). pROC: An open-source package for R and S+ to analyze and compare ROC curves. BMC Bioinf..

[44] Litchfield K., Turajlic S., Swanton C. (2017). The GENIE Is Out of the Bottle: Landmark Cancer Genomics Dataset Released. Cancer Discov..

[45] Zhou W., Chen T., Chong Z., Rohrdanz M.A., Melott J.M., Wakefield C., Zeng J., Weinstein J.N., Meric-Bernstam F., Mills G.B. (2015). TransVar: A multilevel variant annotator for precision genomics. Nat. Methods.

[46] Anagnostopoulos C., Hand D.J., Adams N.M. Measuring Classification Performance: The Hmeasure Package.

[47] Fisher R., Pusztai L., Swanton C. (2013). Cancer heterogeneity: Implications for targeted therapeutics. Br. J. Cancer.

[48] Gore M.E., Larkin J.M.G. (2011). Challenges and opportunities for converting renal cell carcinoma into a chronic disease with targeted therapies. Br. J. Cancer.

[49] Wei P.J., Zhang D., Li H.T., Xia J., Zheng C.H. (2017). Driverfinder: A Gene Length-Based Network Method to Identify Cancer Driver Genes.

[50] Yap T.A., Gerlinger M., Futreal P.A., Pusztai L., Swanton C. (2012). Intratumor Heterogeneity: Seeing the Wood for the Trees. Sci. Transl. Med..

[51] Yang F., Petsalaki E., Rolland T., Hill D.E., Vidal M., Roth F.P. (2015). Protein Domain-Level Landscape of Cancer-Type-Specific Somatic Mutations. PLoS Comput. Biol..

