# Supplementary material for: Identifying Cancer Drivers Using DRIVE: A Feature-Based Machine Learning Model for a Pan-Cancer Assessment of Somatic Missense Mutations
Source: Cancers (Basel). 2021 Jun 3;13(11):2779. doi: 10.3390/cancers13112779 (PMC8199862; doi:10.3390/cancers13112779)
Supplement: Supplementary file 1 [file cancers-13-02779-s001.zip › cancers-1170708-supplementary/Figure S1. Detailed overview of the computational workflow.pdf]

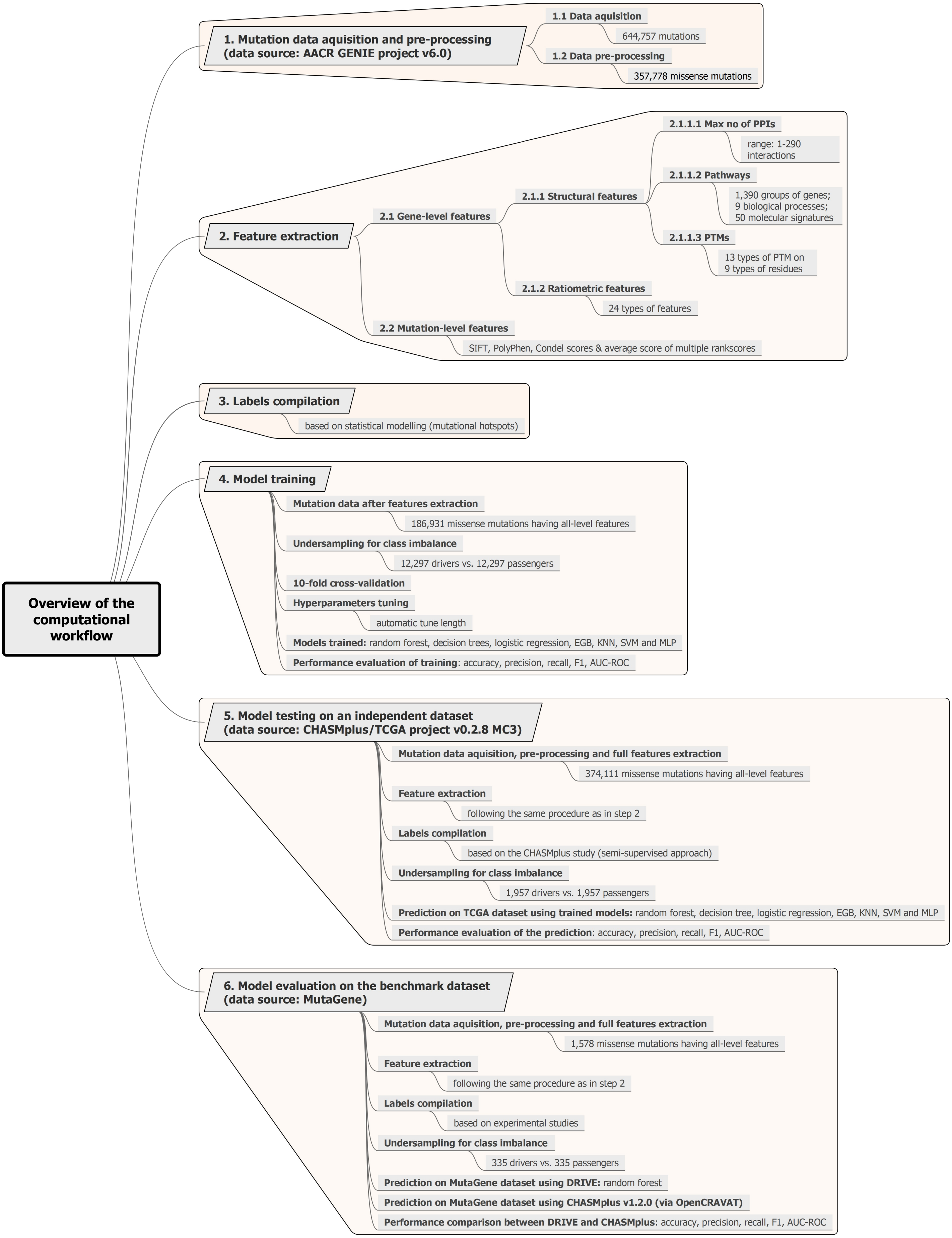
Overview of the computational workflow
1. Mutation data aquisition and pre-processing (data source: AACR GENIE project v6.0)
1.1 Data aquisition
644,757 mutations
1.2 Data pre-processing
357,778 missense mutations
2. Feature extraction
2.1 Gene-level features
2.1.1 Structural features
2.1.1.1 Max no of PPIs
range: 1-290 interactions
2.1.1.2 Pathways
1,390 groups of genes; 9 biological processes; 50 molecular signatures
2.1.1.3 PTMs
13 types of PTM on 9 types of residues
2.1.2 Ratiometric features
24 types of features
2.2 Mutation-level features
SIFT, PolyPhen, Condel scores & average score of multiple rankscores
3. Labels compilation
based on statistical modelling (mutational hotspots)
4. Model training
Mutation data after features extraction
186,931 missense mutations having all-level features
Undersampling for class imbalance
12,297 drivers vs. 12,297 passengers
10-fold cross-validation
Hyperparameters tuning
automatic tune length
Models trained: random forest, decision trees, logistic regression, EGB, KNN, SVM and MLP
Performance evaluation of training: accuracy, precision, recall, F1, AUC-ROC
5. Model testing on an independent dataset (data source: CHASMplus/TCGA project v0.2.8 MC3)
Mutation data aquisition, pre-processing and full features extraction
374,111 missense mutations having all-level features
Feature extraction
following the same procedure as in step 2
Labels compilation
based on the CHASMplus study (semi-supervised approach)
Undersampling for class imbalance
1,957 drivers vs. 1,957 passengers
Prediction on TCGA dataset using trained models: random forest, decision tree, logistic regression, EGB, KNN, SVM and MLP
Performance evaluation of the prediction: accuracy, precision, recall, F1, AUC-ROC
6. Model evaluation on the benchmark dataset (data source: MutaGene)
Mutation data aquisition, pre-processing and full features extraction
1,578 missense mutations having all-level features
Feature extraction
following the same procedure as in step 2
Labels compilation
based on experimental studies
Undersampling for class imbalance
335 drivers vs. 335 passengers
Prediction on MutaGene dataset using DRIVE: random forest
Prediction on MutaGene dataset using CHASMplus v1.2.0 (via OpenCRAVAT)
Performance comparison between DRIVE and CHASMplus: accuracy, precision, recall, F1, AUC-ROC
